# Clinical Significance and Immunometabolism Landscapes of a Novel Recurrence-Associated Lipid Metabolism Signature In Early-Stage Lung Adenocarcinoma: A Comprehensive Analysis

**DOI:** 10.3389/fimmu.2022.783495

**Published:** 2022-02-10

**Authors:** Mingchuang Zhu, Qingpeng Zeng, Tao Fan, Yuanyuan Lei, Feng Wang, Sufei Zheng, Xinfeng Wang, Hui Zeng, Fengwei Tan, Nan Sun, Qi Xue, Jie He

**Affiliations:** ^1^ Department of Oncology, Renmin Hospital of Wuhan University, Wuhan, China; ^2^ National Cancer Center/National Clinical Research Center for Cancer/Cancer Hospital, Chinese Academy of Medical Sciences and Peking Union Medical College, Beijing, China

**Keywords:** lipid metabolism, early-stage lung adenocarcinoma (LUAD), recurrence, immune checkpoints (ICP), signature

## Abstract

**Background:**

The early-stage lung adenocarcinoma (LUAD) rate has increased with heightened public awareness and lung cancer screening implementation. Lipid metabolism abnormalities are associated with lung cancer initiation and progression. However, the comprehensive features and clinical significance of the immunometabolism landscape and lipid metabolism-related genes (LMRGs) in cancer recurrence for early-stage LUAD remain obscure.

**Methods:**

LMRGs were extracted from Gene Set Enrichment Analysis (GSEA) and Kyoto Encyclopedia of Genes and Genomes (KEGG) databases. Samples from The Cancer Genome Atlas (TCGA) were used as training cohort, and samples from four Gene Expression Omnibus (GEO) datasets were used as validation cohorts. The LUAD recurrence-associated LMRG molecular pattern and signature was constructed through unsupervised consensus clustering, time-dependent receiver operating characteristic (ROC), and least absolute shrinkage and selection operator (LASSO) analyses. Kaplan-Meier, ROC, and multivariate Cox regression analyses and prognostic meta-analysis were used to test the suitability and stability of the signature. We used Gene Ontology (GO), KEGG pathway, immune cell infiltration, chemotherapy response analyses, gene set variation analysis (GSVA), and GSEA to explore molecular mechanisms and immune landscapes related to the signature and the potential of the signature to predict immunotherapy or chemotherapy response.

**Results:**

First, two LMRG molecular patterns were established, which showed diverse prognoses and immune infiltration statuses. Then, a 12-gene signature was identified, and a risk model was built. The signature remained an independent prognostic parameter in multivariate Cox regression and prognostic meta-analysis. In addition, this signature stratified patients into high- and low-risk groups with significantly different recurrence rates and was well validated in different clinical subgroups and several independent validation cohorts. The results of GO and KEGG analyses and GSEA showed that there were differences in multiple lipid metabolism, immune response, and drug metabolism pathways between the high- and low-risk groups. Further analyses revealed that the signature-based risk model was related to distinct immune cell proportions, immune checkpoint parameters, and immunotherapy and chemotherapy response, consistent with the GO, KEGG, and GSEA results.

**Conclusions:**

This is the first lipid metabolism-based signature for predicting recurrence, and it could provide vital guidance to achieve optimized antitumor for immunotherapy or chemotherapy for early-stage LUAD.

## Introduction

As the leading cause of cancer-related incidence and mortality, lung cancer accounts for approximately 20% of global cancer-specific deaths (1). Lung adenocarcinoma (LUAD) is the most common pathologic type, accounting for almost 40% of all lung cancer subtypes, and is characterized by rapid progression, severe prognosis, and early recurrence (2). In recent decades, the development of molecular targeted therapy and immune checkpoint inhibitors (ICIs) has to a certain extent improved patient survival in LUAD. However, the overall survival (OS) of LUAD patients remains unfavorable, with a 5-year OS rate of 19% (3). Moreover, even for early-stage LUAD disease, the recurrence rate remains 30–45% within 5 years after surgery (4, 5). The rate of early-stage LUAD has increased rapidly with heightened public awareness and implementation of lung cancer screening (6). Therefore, there is an urgent need to identify those patients with high-risk early-stage LUAD who are likely to experience recurrence to optimize personalized therapeutic strategies and improve patient survival.

As a unique metabolic niche, the tumor microenvironment (TME) contains cellular components (tumor cells, immune cells, and stromal cells) and the tumor interstitial space. Because of high proliferation and inadequate angiogenesis, tumor cells reprogram their energy metabolism in the TME (7, 8). In recent years, lipid metabolism has been reported to be a potential hallmark in multiple malignancies (9–11). Tumor cells are characterized by excess lipid and cholesterol uptake, and upregulated uptake promotes the proliferation and division of tumor cells (12). Moreover, lipid metabolism reprogramming may also act as a potential pathway for drug resistance in antitumor therapy (13). An increasing number of studies have focused on the role of lipid-related phenotypic indices in various cancers. Ding et al. (14) reported that a specific fatty acid metabolism-related gene signature could predict patient survival and response to chemotherapy and immunotherapy in colorectal cancer. Wu et al. (15) also described a lipid metabolism-related phenotype and constructed a lipid metabolic gene signature to predict patient survival in diffuse gliomas. However, the features of lipid metabolism alteration and whether it has the potential to be a biomarker for cancer recurrence and treatment response in early-stage LUAD patients still warrant further exploration.

In this study, a series of bioinformatic methods were applied to analyze the features of lipid metabolism alterations in early-stage LUAD based on transcriptional profiling data from multiple databases. Then, a lipid metabolism-related gene signature for predicting cancer recurrence was established and validated. Moreover, we also investigated the differences in lipid metabolism and immune landscapes between the low- and high-risk groups. Finally, the potential of our signature as a biomarker to predict immunotherapy and chemotherapy response in early-stage LUAD patients was also investigated. Thus, this study will be helpful for promoting individualized treatment and reducing the postoperative recurrence rates of early-stage LUAD patients.

## Materials and Methods

### Lipid Metabolism-Related Genes

Lipid metabolism-related genes (LMRGs) were selected through the Gene Set Enrichment Analysis (GSEA) database and Kyoto Encyclopedia of Genes and Genomes (KEGG) database. A total of 1133 LMRGs were extracted from 19 lipid metabolism-related gene sets of the GSEA database, and 426 LMRGs were extracted from 16 lipid metabolism-related gene sets of the KEGG database. The detailed gene sets from the GSEA and KEGG databases are shown in **Table S1**. After removing the duplicate genes, a total of 1189 LMRGs were identified for further investigation.

### Patient and mRNA Data

A total of 805 cases of early-stage (stage I-II) LUAD with corresponding recurrence-free survival (RFS) data from five independent cohorts were included for analysis. The clinical characteristics of the five cohorts are shown in **Table 1**. The 334 LUAD cases from The Cancer Genome Atlas (TCGA) database (https://portal.gdc.cancer.gov/) were used as the training cohort, whereas the 471 LUAD cases from four Gene Expression Omnibus (GEO) datasets (http://www.ncbi.nlm.nih.gov/geo) were applied as the validation cohort (including 226 cases from GSE31210, 81 cases from GSE30219, 121 cases from GSE50081, and 43 cases from GSE37745). TCGA RNA-seq data of the enrolled LUAD cases (Illumina HiSeq 2000) with detailed clinical annotations and survival data were acquired. The mRNA expression data from the GEO microarray were first log2 transformed and quantile normalized, and the mean expression was selected if the genes were detected with more than one probe. Patients with low levels of gene expression or less than three months of survival and follow-up time were excluded.

**Table 1:** Clinical characteristics of the patients from multiple institutions.

Characteristics		Datasets				
		TCGA (n = 334)	GSE31210 (n = 226)	GSE30219 (n = 81)	GSE50081 (n = 121)	GSE37745 (n = 43)
Age						
	<=65	156	176	59	40	20
	>65	169	50	22	81	23
	unknown	9				
Sex						
	male	153	105	64	62	18
	female	181	121	17	59	25
Smoking						
	no	50	115		22	
	yes	275	111		88	
	unknown	9			11	
EGFR						
	wild	290	99			
	mutation	38	127			
	unknown	6				
KRAS						
	wild	248	206			
	mutation	80	20			
	unknown	6				
ALK						
	wild	306	215			
	mutation	22	11			
	unknown	6				
T stage						
	T1	128		68	41	
	T2	181		12	78	
	T3	25		1	2	
N stage						
	N0	264		79	90	
	N1	65		2	31	
	unknown	5				
Stage						
	stage I	232	168		88	33
	stage II	102	58		33	10
OS status						
	dead	101	35	42	49	27
	alive	233	191	39	72	16
Recurrence status						
	recurrence	127	64	26	37	21
	no-recurrence	207	162	55	84	22

### LMRG Molecular Patterns

Univariate Cox regression was applied to identify recurrence-associated LMRGs, and genes with a *P* value less than 0.05 were selected. Unsupervised consensus clustering was applied to investigate the molecular classification of LUAD according to the recurrence-associated LMRGs by using the “ConsensusClusterPlus” R package (16), with 1000 iterations to improve the stability. Then, comparisons were performed within different clusters for the survival and tumor immune microenvironment (TIM) analyses.

### Signature Construction

We also attempted to construct an LMRG-based signature for recurrence prediction in early-stage LUAD patients. Time-dependent receiver operating characteristic (ROC) analysis was used to evaluate the correlation between recurrence-associated LMRGs and RFS, and genes with an area under the curve (AUC) less than 0.60 were removed. We then performed least absolute shrinkage and selection operator (LASSO) analysis to identify significant prognostic LMRGs and create a recurrence risk score model based on the LMRGs. Finally, a risk score model was constructed by taking into account the expression value of optimized genes and the estimated Cox regression correlation coefficients: $\text{Risk score}=\Sigma_{1}^{i}coeffi*expGenei$ . All enrolled patients were then classified into high- and low-risk groups according to the median value of the given risk scores calculated from the TCGA cohort. Kaplan-Meier analysis, ROC analysis, multivariate Cox regression analysis, and prognostic meta-analysis were used to test the suitability and stability of the model.

### Pathway and Functional Enrichment Analyses

For biological process and pathway enrichment analyses, KEGG and Gene Ontology (GO) analyses were performed by R clusterProfiler package. Gene set enrichment analysis (GSEA) was performed using GSEA software (version 4.1.0).

### Tumor Immune Microenvironment Analysis

The CIBERSORT (17) algorithm was employed to quantify the proportions and distributions of tumor-infiltrating immune cells (TIICs) based on the RNA-seq data of TCGA specimens. The LM22 signature algorithm was utilized to calculate the abundances of 22 types of TIICs. We also used the ESTIMATE (18) algorithm to evaluate the immune and stromal scores (reflecting the abundances of immune cells and stromal cells, respectively) for each lung cancer sample.

### GSVA

We also performed gene set variation analysis (GSVA) (19) to evaluate the correlation between seven clusters of inflammatory and immune response metagenes and the lipid metabolism-based signature.

### Immune Checkpoint Profile Analysis

The somatic mutation data were also downloaded from TCGA database. Tumor mutation burden (TMB), a potential biomarker for immunotherapy response, was calculated based on somatic nonsynonymous mutations. The expression levels of PD-L1 were also extracted and assessed. Both TMB and PD-L1 level are widely used biomarkers for the efficacy evaluation of ICIs (20, 21). Moreover, tumor immune dysfunction and exclusion (TIDE), a computational algorithm that can evaluate the signatures of T cell dysfunction (22), was also applied to predict the clinical response to immunotherapy of LUAD patients based on expression profiles. The TIDE score has been reported to show superior efficiency in the prediction of anti-PD1 or anti-CTLA4 therapy response compared with the biomarkers TMB and PD-L1 level (22). In addition, as another biomarker of immunotherapy response, tumor-specific neoantigen data were also obtained from The Cancer Immunome Atlas (TCIA) and analyzed (23, 24).

### Chemotherapeutic Response Prediction

Furthermore, based on the Genomics of Drug Sensitivity in Cancer (GDSC) database, we performed chemotherapeutic response prediction for each LUAD sample. Four commonly used drugs were selected, namely, cisplatin, docetaxel, doxorubicin, and gemcitabine. The R package “pRRophetic” (25) was utilized for calculation, and the half-maximal inhibitory concentration (IC_50_) was estimated for each of the above drugs.

### Statistical Analysis

All data analyses were performed by using R 4.0.3 software, SPSS 26.0, and GraphPad Prism 8. Correlation analysis was performed with Pearson’s correlation test. The Kruskal-Wallis test was used to compare more than two groups, and the Wilcoxon test was used to compare two groups. A two-tailed P-value <0.05 was considered statistically significant.

## Results

### Recurrence-Associated LMRG Identification and Clustering

**Figure 1:**
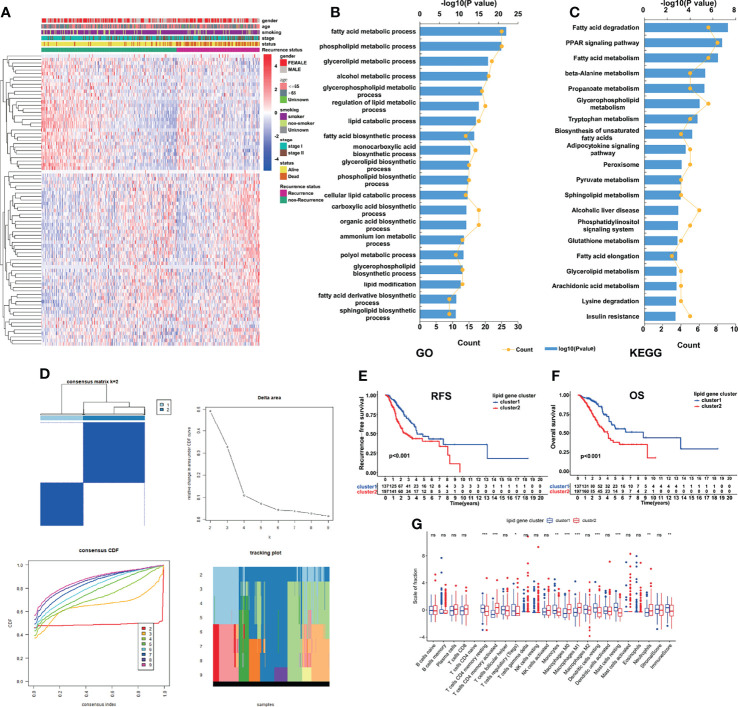
Identification of LMRG molecular patterns with distinct prognoses and immune infiltration statuses. **(A)**, Heatmap of the expression patterns of 83 recurrence-associated LMRGs. **(B, C)**, GO and KEGG analyses of the identified genes. **(D)**, Consensus clustering results, in which the optimal cluster number was 2 (k value=2). **(E, F)**, Kaplan–Meier curve survival analysis of patients stratified by cluster subtype. **(G)**, Immune cell infiltration landscapes and the immune and stromal scores of the two cluster subtypes. *, ** and *** represent P < 0.05, P < 0.01, and P < 0.001, respectively. ns indicates not significant.

**Figure 2:**
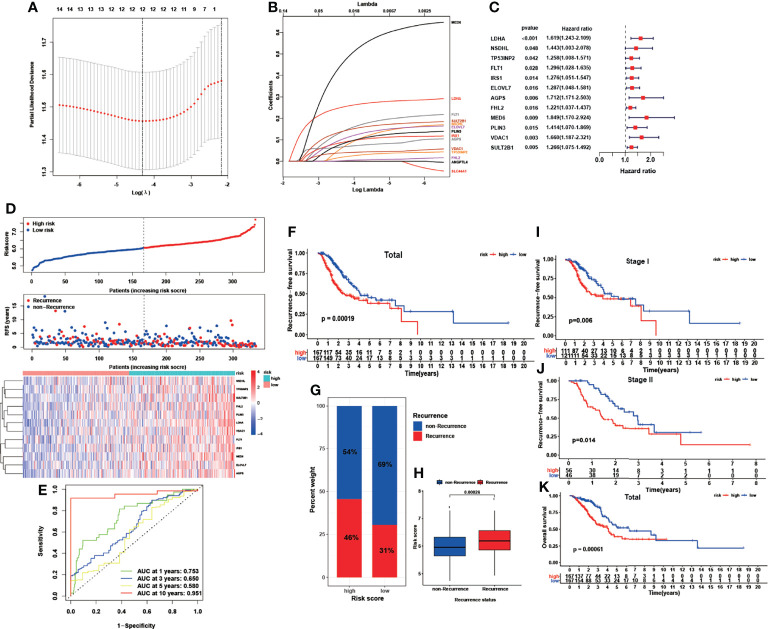
Construction of a recurrence-associated LMRG-based signature for early-stage LUAD. **(A, B)**, LASSO coefficient profile analysis and cross-validation to identify the most useful prognostic genes. **(C)**, Twelve lipid metabolism genes identified for signature construction. **(D)**, Distributions of risk scores, recurrence statuses and gene expression. **(E)**, ROC results of the LMRG-based signature for the prediction of recurrence risk at 1, 3, 5, and 10 years. **(F)** Kaplan-Meier curves of RFS in the TCGA cohort based on risk score. **(G, H)**, Correlation between recurrence status and risk score. **(I, J)**, Kaplan-Meier curves of RFS in stage I and stage II early-stage LUAD based on risk score. **(K)**, Kaplan-Meier curves of OS in the TCGA cohort based on risk score.

The overall workflow of the current study is displayed in **Figure S1**. Based on 334 early-stage LUAD patients from the TCGA database, we performed univariate Cox regression analysis and found that 83 LMRGs were significantly associated with cancer recurrence (**Table S2**, *p*<0.05). **Figure 1A** shows the heatmap of the expression patterns of the 83 LMRGs. The GO and KEGG biological process and pathway enrichment analyses showed that these significant LMRGs were primarily involved in the fatty acids biosynthetic process and other lipid metabolic pathways (**Figures 1B, C**). Unsupervised consensus clustering based on the expression patterns of the 83 recurrence-associated LMRGs revealed the optimal number of clusters to be two (k value=2) (**Figure 1D**). Hence, all 334 LUAD patients were divided into two subsets, cluster 1 (137, 41.0%) and cluster 2 (197, 59.0%). The survival analyses showed that patients in the cluster 2 group showed a significantly inferior RFS and OS rate compared with those in the cluster 1 group (**Figure 1E**, *P*<0.001). There were several recurrence-associated LMRGs which were not detected in the validation groups. However, although the number of genes missing from the validation group was small, we still performed this analysis on the validation groups by their optimal k values separately. The results showed that the recurrence-associated LMRGs could divide patients into different gene cluster groups. There were also significant differences in both RFS and OS between different gene cluster groups (FigS2). These results revealed that the recurrence-associated LMRGs were indeed closely related to the recurrence and long-term survival in early-stage LUAD. Through CIBERSORT and ESTIMATE analyses, we quantified the abundance of 22 types of TIICs and the immune and stromal scores in every LUAD sample of the TCGA cohort. Our results showed that resting memory CD4 T cells, regulatory T cells (Tregs), monocytes, resting dendritic cells, and resting mast cells were more abundant in cluster 1 patients (**Figure 1G**, P<0.05), whereas activated memory CD4 T cells, M0 macrophages, M1 macrophages, and neutrophils were more abundant in cluster 2 patients (**Figure 1G**, *P*<0.05). Moreover, the immune score was significantly higher, indicating a higher abundance of infiltrating immune cells, in the patients of cluster 1 (**Figure 1G**, *P*<0.05). However, no significant difference in the stromal score between the two subsets was observed.

### LMRG-Based Signature Construction

In the subsequent ROC analysis, we calculated the corresponding AUC value of each of the 83 LMRGs to filter them, and 69 genes with an AUC value less than 0.6 were screened out. Finally, we applied LASSO regression analysis to identify the most powerful LMRGs, and a final set of 12 genes (*LDHA, NSDHL, TP53INP2, FLT1, IRS1, ELOVL7, AGPS, FHL2, MED6, PLIN3, VDAC1*, and *SULT2B1*) was selected for model construction (**Figures 2A–C**). The recurrence risk score of each sample was calculated with the following formula: Risk score = (0.269752653768969 * LDHA) + (0.114984371234971 * NSDHL) + (0.0310569577143764 * TP53INP2) + (0.17593670859331 * FLT1) + (0.103773854013971 * IRS1) + (0.133274629300893 * ELOVL7) + (0.0763835280754936 * AGPS) + (0.00688026723004499 * FHL2) + (0.549703365193899 * MED6) + (0.109916205934083 * PLIN3) + (0.0437103013689035 * VDAC1) + (0.160609136698149 * SULT2B1).

### Prognostic Significance of the LMRG-Based Signature

All the patients in the TCGA cohort were classified into low- and high-risk groups according to the median cutoff value of the given risk scores (**Figure 2D**). The detailed expression levels of the 12 genes between the two groups are also shown in **Figure 2D**. The performance of this signature was evaluated through time-dependent ROC curves, and the AUCs were 0.753, 0.650, 0.580, and 0.951 for predicting 1-, 3-, 5-, and 10-year RFS, respectively (**Figure 2E**). In addition, patients in the high-risk group showed an inferior RFS rate (**Figure 2F**, *P*<0.001) and a higher recurrence rate (**Figure 2G**, *P*=0.004) than those in the low-risk group. Patients who experienced cancer recurrence also showed a significantly elevated risk score (**Figure 2H**, *P*<0.001). In subgroups stratified by TNM stage, similar results were found: the high-risk patients with either stage I (**Figure 2I**, *P*=0.006) or stage II disease (**Figure 2J**, *P*=0.014) showed a poorer RFS rate than low-risk patients. In addition to RFS, patients in the high-risk group similarly showed an inferior OS rate compared with those in the low-risk group (**Figure 2K**, *P*<0.001).

The performance of this predictive model was further evaluated and well validated in subgroups stratified by age, sex, smoking status, and driven gene mutation status. As illustrated in **Figure S3**, nearly all subgroups exhibited a significantly impaired RFS rate in the high-risk group compared with the low-risk group (*P*<0.05), which verified the robust discriminatory ability of the risk model. Although not significant, patients in the high-risk group in the nonsmoker (*P=*0.081) and ALK-wild-type subgroups (*P=*0.053) similarly showed a lower RFS rate than those in the low-risk group, which could be due to the limited sample sizes of these subgroups.

### Signature Validation in GEO Datasets

**Figure 3:**
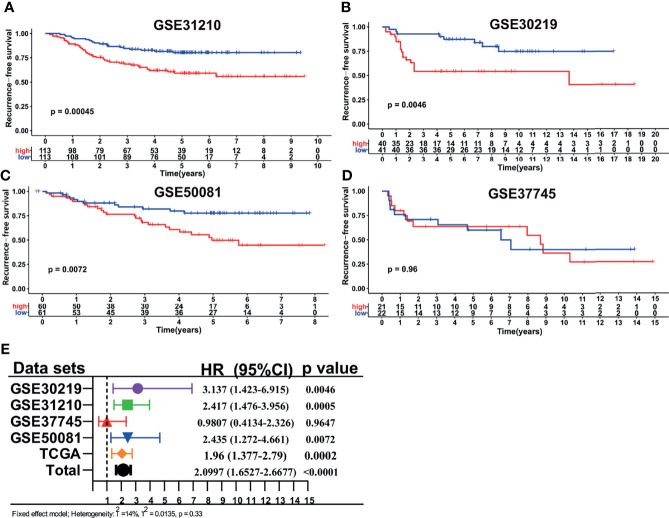
Validation of the LMRG-based signature in different GEO cohorts. **(A–D)**, Kaplan-Meier curves of RFS in different GEO cohorts based on risk score. **(E)**, Results of the prognostic meta-analysis based on the TCGA and GEO datasets.

The other four independent cohorts from the GEO database were utilized as the validation cohorts to evaluate the performance of our LMRG-based signature. The risk scores for the validation cohorts were generated, and patients were divided into high- and low-risk groups using the median cutoff value of the generated risk scores. The survival analysis (**Figure 3**) revealed that patients in the high-risk group showed an inferior RFS compared with those in the low-risk group in GSE31210 (*P=*0.0004), GSE30219 (*P=*0.0046), and GSE50081 (*P=*0.0072). However, in the GSE37745 cohort, patients in the low- and high-risk groups showed a similar RFS rate, which may be due to the small sample size of the two cohorts. Moreover, we conducted a prognostic meta-analysis to evaluate the comprehensive predictive value of our model in all five cohorts. The results revealed that the LMRG-based signature was a significant predictor of cancer recurrence in early-stage LUAD (**Figure 3**, HR = 2.09, 95%CI: 1.65–2.66, *P* < 0.0001). The prognostic meta-analysis was also conducted in subgroups stratified by TNM stage. The GSE30219 cohort was not included in the subanalysis because stage information was unavailable. As shown in **Figure S4**, the LMRG-based signature was similarly revealed as a significant predictor of cancer recurrence in stage I disease (HR = 2.08, 95%CI: 1.50–2.89, *P* < 0.0001) and stage II disease (HR = 1.56, 95%CI: 1.03–2.35, *P* = 0.0345).

The ability of our LMRG-based signature to predict OS was also investigated. The survival analysis (**Figure S5**) revealed that patients in the high-risk group showed an inferior OS than those in the low-risk group in GSE31210 (*P=*0.0003) and GSE30219 (*P=*0.0015), whereas in the GSE37745 (*P*=0.46) and GSE50081 (*P=*0.29) cohorts, the survival difference between the two groups failed to reach a significant level. The final prognostic meta-analysis similarly revealed our LMRG-based signature to be a significant predictor of OS in early-stage LUAD (**Figure S5E**, HR = 2.00, 95%CI: 1.56–2.57, *P* < 0.0001). Thus, the above results altogether verified the robustness and universality of our signature.

### The LMRG-Based Signature Was an Independent Predictor of Patient Survival

Since the LMRG-based signature had been well validated in the other independent cohorts, we carried out univariate and multivariate Cox regression analyses to evaluate whether this signature could be an independent predictor of the prognosis of early-stage LUAD patients. Our results showed that the risk score remained an independent indicator of unfavorable RFS (**Table 2**, HR=1.923, 95%CI: 1.445–2.986, *P*<0.001) and OS (**Table 2**, HR=1.935, 95%CI: 1.287–2.909, *P*=0.002) after adjusting for other clinical parameters (including age, sex, smoking history, driver gene mutations, and TNM stage).

**Table 2:** Univariable and multivariable Cox regression analysis of the LMRG-based signature and survival in TCGA dataset.

Variables		Ref.	Univariate analysis		Multivariate analysis	
			HR (95% CI)	*P* value	HR (95% CI)	*P* value
**RFS**						
Age	≤65/>65	≤65	1.298 (0.907 - 1.858)	0.153		
	Unknown		0.682 (0.270 - 1.724)	0.419		
Sex	male/female	male	1.128 (0.792 - 1.605)	0.504		
Smoking	No/Yes	No	1.075 (0.650 - 1.777)	0.778		
	Unknown		1.036 (0.384 - 2.798)	0.944		
EGFR	Wild/Mutation	Wild	1.710 (1.046 - 2.795)	0.032		
	Unknown		0.839 (0.252 - 2.792)	0.774		
KRAS	Wild/Mutation	Wild	0.889 (0.584 - 1.353)	0.582		
	Unknown		0.767 (0.229 - 2.575)	0.668		
ALK	Wild/Mutation	Wild	1.345 (0.682 - 2.655)	0.392		
	Unknown		0.812 (0.245 - 2.694)	0.734		
T stage	T1/T2	T1	1.515 (1.026 - 2.237)	0.037		
	T3		2.710 (1.408 - 5.217)	0.003		
N stage	N0/N1	N0	1.643 (1.097 -2.461)	0.016		
	Unknown		–	0.964		
Stage	I/II	I	2.116 (1.471 - 3.044)	<0.001	2.077 (1.445 - 2.986)	<0.001
Risk score	Low/High	Low	1.955 (1.366 - 2.798)	<0.001	1.923 (1.343 - 2.752)	<0.001
**OS**						
Age	≤65/>65	≤65	1.401 (0.939 - 2.091)	0.099		
	Unknown		0.302 (0.073 - 1.260)	0.1		
Sex	male/female	male	0.988 (0.667 - 1.463)	0.952		
Smoking	No/Yes	No	1.036 (0.595 - 1.801)	0.902		
	Unknown		1.075 (0.388 - 2.979)	0.889		
EGFR	Wild/Mutation	Wild	1.518 (0.875 - 2.632)	0.137		
	Unknown		0.523 (0.114 - 2.393)	0.403		
KRAS	Wild/Mutation	Wild	0.862 (0.535 - 1.390)	0.543		
	Unknown		0.475 (0.102 - 2.204)	0.342		
ALK	Wild/Mutation	Wild	1.037 (0.480 - 2.238)	0.927		
	Unknown		0.503 (0.111 - 2.290)	0.374		
T stage	T1/T2	T1	1.530 (0.982 - 2.386)	0.06		
	T3		2.099 (0.949 - 4.643)	0.067		
N stage	N0/N1	N0	2.383 (1.563 - 3.633)	<0.001		
	Unknown		0.900 (0.124 - 6.507)	0.917		
Stage	I/II	I	2.868 (1.917 - 4.290)	<0.001	2.785 (1.862 - 4.165)	<0.001
Risk score	Low/High	Low	2.011 (1.338 - 3.022)	0.001	1.935 (1.287 - 2.909)	0.002

### Biological Pathways and Functional Enrichment Analysis

**Figure 4:**
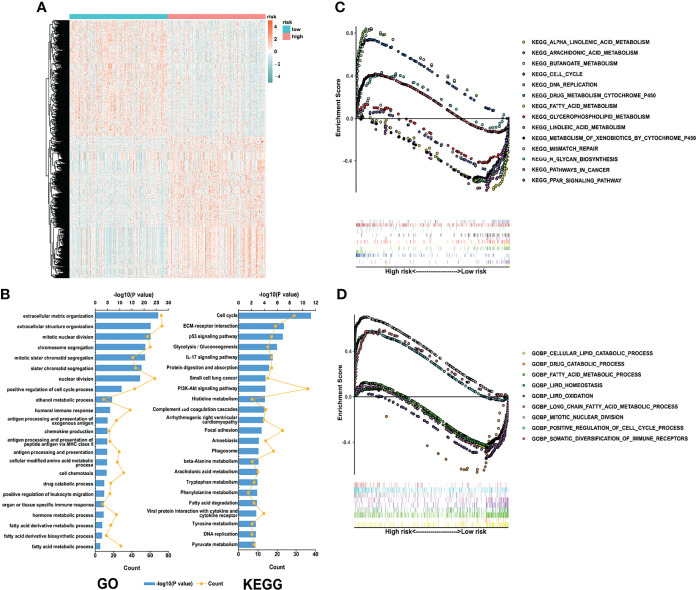
Biological process and pathway analyses of the LMRG-based signature. **(A)**, Heatmap of the differentially expressed genes between the two risk groups. **(B)**, GO and KEGG analyses of the identified genes. **(C, D)**, Representative pathways enriched in the identified genes as determined by GSEA (normal p value<0.05).

Given the satisfying prognostic performance of our LMRG-based signature in early-stage LUAD patients, we investigated the underlying mechanism. First, differential expression analysis was conducted between the two risk groups (|log2 fold change|>=0.5, adjusted P value<0.05). The results showed that 547 genes were overexpressed and 445 genes were expressed at lower levels in the high-risk group (**Figure 4A**). Then, GO and KEGG analyses of these DEGs were conducted. The biological process analysis showed that the DEGs were enriched in multiple extracellular matrix organization, cell division, metabolism and immune response pathways (**Figure 4B**). Moreover, GSEA was performed to further explore the most significantly enriched functional terms between the high-risk and low-risk patients. We found that the cell cycle was enriched in the high-risk group. However, drug metabolism and fatty acid metabolism were enriched in the low-risk patients (**Figures 4C, D**). The above results indicated that immune activity and drug metabolism are potential mechanisms underlying the ability of our LMRG-based signature to predict the prognosis of early-stage LUAD patients.

### Immune and Inflammatory Landscapes Related to the LMRG-Based Signature

**Figure 5:**
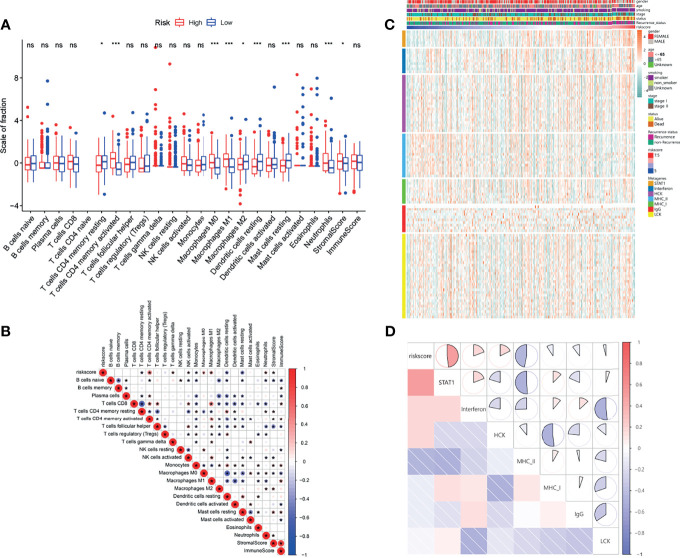
Immune and inflammatory landscapes related to the LMRG-based signature. **(A)**, Immune cell infiltration levels and immune and stromal scores of the two risk groups. **(B)**, Correlation heatmap showing the interaction between estimated immune cell infiltration levels and risk score. **(C)**, Relationships between risk score and seven clusters of inflammatory activity-related metagenes in the TCGA cohort. **(D)**, Corrgrams showing the correlations between risk score and seven metagenes based on the Pearson r value. * and *** represent P < 0.05 and P < 0.001, respectively. ns indicates not significant.

As indicated by the above results, the LMRG-based signature was closely correlated with tumor immune activities. As such, we next evaluated the differential levels of specific immune characteristics, including the abundance of TIICs. Our results showed that resting memory CD4 T cells, M2 macrophages, resting dendritic cells, and resting mast cells were more abundant in patients in the low-risk group (**Figure 5A**, *P*<0.05), whereas activated memory CD4 T cells, M0 macrophages, M1 macrophages, and neutrophils were more abundant in patients in the high-risk group (**Figure 5A**, *P*<0.05). In addition, the high-risk patients also showed relatively higher stromal and immune scores (**Figure 5A**), indicating higher abundances of stromal cells and infiltrating immune cells, respectively. Moreover, correlation analysis was also performed between the risk score and the differential TIICs. The results showed that the risk score was positively correlated with activated memory CD4 T cells, M0 macrophages, M1 macrophages, and neutrophils but negatively correlated with resting memory CD4 T cells, resting dendritic cells, and resting mast cells (**Figure 5B**).

In addition, a seven-metagene cluster (STAT1, interferon, HCK, MHC-I, MHC-II, IgG, and LCK) has been reported in several studies (26–28) to illustrate the inflammatory activities in the tumor microenvironment. Hence, we analyzed the correlation between the risk score and this seven-metagene cluster. The GSVA package was utilized to evaluate the molecular pathway variation associated with the seven-metagene set. The detailed expression levels of the genes in the seven-metagene cluster are shown in **Figure 5C**. To better illustrate the correlation, a correlogram was employed. Our results indicated that the risk score was positively correlated with STAT1, interferon, and HCK but negatively correlated with MCH-II (**Figure 5D**).

### Immune Checkpoint Profile and Immunotherapy Response Prediction

**Figure 6:**
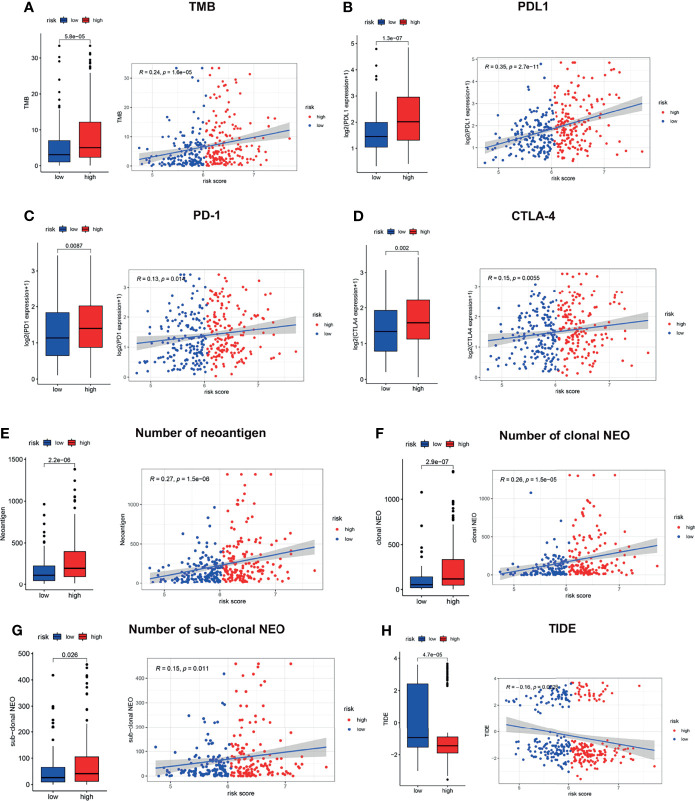
Immune checkpoint profile related to the LMRG-based signature. The estimated TMB **(A)**, PD-L1 **(B)**, PD-1 **(C)**, CTLA-4 **(D)**, neoantigen burden **(E–G)** and TIDE score **(H)** in the two risk groups are shown.

Since the LMRG-based signature was revealed to be associated with immune activity, we next evaluated the immune checkpoint profile and conducted a preliminary analysis of immunotherapy response. The TMB has been well studied as a biomarker of response to checkpoint inhibitors, and high TMB patients may be more likely to benefit from ICIs in NSCLC (29). As illustrated in **Figure 6A**, patients in the high-risk group showed a significantly higher TMB than those in the low-risk group (*P*<0.001). In addition, somatic mutation analysis was applied to explore the distinct genomic variations between the two groups. Patients in the high-risk group showed an elevated mutation rate compared with those in the low-risk group (**Figure S6**, 92.8% *vs*. 88.2%). Tumorigenesis-associated genes, including TP53, TNN, and MUC16, showed a much higher mutation rate in the high-risk group than in the low-risk group (**Figure S6**). We also found that PD-L1, PD-1, and CTLA-4 showed a distinct expression level between the high-risk and low-risk groups (**Figures 6B–D**, *P*<0.01), which was consistent with the results of the TMB analysis. Besides, patients in the high-risk group also showed a significantly higher LAG3 and TIM3 than those in the low-risk group (**Figures S7A, S7B**, *P*<0.01). Neoantigens play a vital role in the antitumor response (30), and previous research has revealed that neoantigens could be a biomarker for predicting immunotherapy response in lung cancer (31). Our results showed that the high-risk patients had a significantly elevated number of neoantigens (**Figure 6E**, *P*<0.001), both clonal (**Figure 6F**, *P*<0.001) and subclonal (**Figure 6G**, *P*<0.05) neoantigens. The TIDE score, indicating the potential for tumor immune evasion, has shown superior immunotherapy response prediction compared with TMB, PD-L1 level, and neoantigen burden (22). We revealed that high-risk patients showed a significantly decreased TIDE score (**Figure 6H**, *P*<0.01).

Moreover, we found that high-risk patients showed a significantly decreased tumor-associated macrophage (TAM) M2 score (**Figure S7C**, *P*<0.001) and a significantly increased myeloid-derived suppressor cell (MDSC) score (**Figure S7D**, *P*<0.001), cancer-associated fibroblast (CAF) score (**Figure S7E**, *P*<0.05), and CD8 score (**Figure S7F**, *P*<0.001). Moreover, correlation analysis was also carried out, and the risk score was found to be positively correlated with T cell exclusion (**Figure S7G**, *P*<0.01) but negatively correlated with T cell dysfunction (**Figure S7H**, *P*=0.0015). Hence, we can conclude that high-risk patients are likely to benefit from the administration of checkpoint inhibitors. These interesting findings demonstrated that the high- and low-risk patients had diverse immune statuses, and our LMRG-based signature could identify those who were suitable for treatment with checkpoint inhibitors.

### Chemotherapy Response Prediction

**Figure 7:**
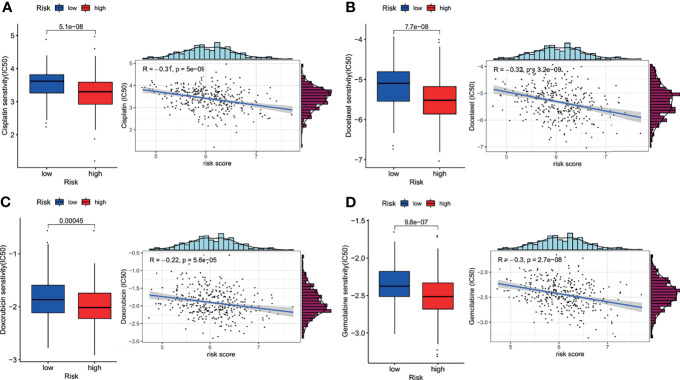
Impact of risk score on chemotherapy response. The estimated half-maximal inhibitory concentration (IC_50_) of cisplatin **(A)**, docetaxel **(B)**, doxorubicin **(C)**, and gemcitabine **(D)** for response between the low- and high-risk groups.

Since the pathway analysis revealed that low-risk patients showed enrichment of drug metabolism, we carried out chemotherapy response prediction. The four conventionally used drugs for NSCLC were used for the analysis. The results showed that the estimated IC_50_ for each of the four agents (cisplatin, docetaxel, doxorubicin, and gemcitabine) was significantly higher in the low-risk group than in the high-risk group (**Figure 7**, *P*<0.001), indicating that LUAD patients with lower risk scores tended to be more resistant to chemotherapy than those with higher risk scores.

## Discussion

Metabolic deregulation has been revealed to play an essential role in various malignancies due to its impact on tumor growth, proliferation, invasion, and treatment response (32). For instance, various malignancies are characterized by upregulated glycolytic metabolism (33). Lipids, including fatty acids, cholesterol, and phospholipids, act as essential substances to maintain cytoskeletal structure, provide energy, and participate in cellular signal transduction. Lipid metabolism reprogramming has been revealed to be correlated with membrane synthesis, energy production, and signal transduction, hence impacting the TME, immunity, and drug resistance in multiple malignancies (34, 35). Moreover, aberrant lipid metabolism has also been studied and verified as an important factor involved in lung cancer pathogenesis and progression (36).

Implementation of the current lung cancer screening program has caused the rate of early-stage lung carcinoma diagnosis to increase (6). However, there is still a proportion of early-stage patients who will experience early cancer relapse after the initial curative treatment. Hence, effective methods are needed to identify patients at high risk of cancer recurrence to improving the prognosis of early-stage patients. Among all the prognostic biomarkers, multiple gene-based signatures based on specific biological processes have shown superiority in survival prediction in various malignancies (28, 37, 38). However, to our knowledge, prognostic gene signatures based on lipid metabolism have not been reported in early-stage LUAD.

In this study, for the first time, we investigated the immunometabolism landscape of patients with early-stage LUAD through genetic subtype analysis and with a gene signature-based model. We also evaluated the relationship between the identified LMRGs and the prognosis of early-stage LUAD. Using multiple datasets from the TCGA and GEO cohorts, a 12-gene signature was established as a significant predictor of recurrence and survival. The performance of this signature was also well validated in internal subsets as well as in external independent cohorts. In addition, we also explored the potential molecular mechanism underlying the prognostic value of this signature for early-stage LUAD patients. The results showed that this signature was associated with diverse lipid metabolism and inflammatory and immune pathways, and such pathways may be the mechanism underlying the prognostic value of this signature. Moreover, our results also indicated that this signature was related to different immune checkpoint profiles and drug sensitivities, which verified that this LMRG-based signature could identify patients who were suitable for treatment with immunotherapy or chemotherapy.

In the present study, we first identified 83 recurrence-associated LMRGs through the GSEA and KEGG databases. Based on the mRNA expression profiles of the 83 LMRGs, a two-category lipid metabolism molecular pattern was established using unsupervised consensus clustering for early-stage LUAD patients. We found significant differences in terms of survival and immune infiltration between the two molecular patterns. This finding indicated that lipid metabolism abnormalities may contribute to the aggressiveness and progression of LUAD, hence impacting patient outcomes. The results of the validation groups also showed that these recurrence-associated LMRGs could divide the patients into different gene cluster groups. There were significant differences in prognosis between the different cluster groups. These results suggested that these LMRGs were indeed closely related to recurrence and long-term survival in early-stage LUAD patients. And all these LMRGs selected were scientific and credible. Moreover, the underlying mechanism for this observation may be due to differences in immune activity.

Furthermore, we developed a 12-LMRG recurrence signature using 334 early-stage LUAD patients from the TCGA database. This signature includes *LDHA, NSDHL, TP53INP2, FLT1, IRS1, ELOVL7, AGPS, FHL2, MED6, PLIN3, VDAC1*, and *SULT2B1*, most of which were revealed to be correlated with tumor proliferation and progression (39–47). For instance, NSDHL, a crucial enzyme for cholesterol biosynthesis, has been reported to promote metastasis in triple-negative breast cancer (48). Furthermore, FLT1 was also found to be a potential tumor suppressor, and the hypermethylation of FLT1 may contribute to polycyclic aromatic hydrocarbon-induced carcinogenicity (42). However, the role of *MED6* in malignancies has not yet been reported. Based on our 12-LMRG signature, each early-stage LUAD patient was assigned a risk score, which estimates the probability of cancer recurrence; by so doing, the high-risk patients were effectively identified.

The prognostic performance of this signature for early-stage LUAD patients was well validated in internal subsets. Since there has been increasing detection of stage I LUAD due to low-dose computed tomography (LDCT) screening, we investigated the signature in the subgroups stratified by TNM stage. The results showed that the signature performed well in stage I and stage II disease. In addition, other clinical parameters, namely, age, sex, smoking status, and driven gene mutations, were verified to be risk factors or predictive variables for patient survival in LUAD (49–51). We also evaluated the applicability of the signature in these subgroups. As expected, the signature similarly performed well in the clinical subgroups. The robust discriminatory ability of this signature in subsets of early-stage LUAD patients highlighted its independent value. Moreover, this signature was also well validated in four external independent GEO cohorts. Although the survival difference failed to reach a significant level in the GSE37745 cohort, further prognostic meta-analysis verified this signature to be an independent predictor of prognosis in multiple cohorts. It should be emphasized that the cutoff for determining if patients fell into the high- or low-risk group was the median value of the calculated risk scores in both the training and validation cohorts. Previous signature-based studies usually divide patients using the optimal cutoff value (28, 52); hence, the universality of the developed model will be largely affected.

Since our signature was revealed to be effective for survival prediction across multiple cohorts and subgroups, we aimed to explore the underlying mechanisms. A total of 992 genes were revealed to be correlated with the risk score, and further GO and KEGG analyses suggested that the genes were enriched in multiple biological processes, especially those related to extracellular structure, cell division, lipid metabolism and immune response pathways. Further GSEA showed that different risk groups had significant differences in drug metabolism and fatty acid metabolism. The above results preliminarily explained that the differences in prognosis between the high- and low-risk groups may be attributed to immune activity and drug metabolism.

Based on the above results, we then carried out TIIC analysis and metagene analysis to provide more insight into the immune and inflammatory landscapes of early-stage LUAD. Our results showed that the risk score was positively correlated with STAT1, interferon, and HCK but negatively correlated with MCH-II. Moreover, TIIC analysis showed that patients in the high-risk group were characterized by high proportions of activated memory CD4 T cells, M0 macrophages, M1 macrophages, and neutrophils and low proportions of resting memory CD4 T cells, M2 macrophages, resting dendritic cells, and resting mast cells. The above results suggested that variations in immune and inflammatory activity and TIIC composition may be potential mechanisms that affect the probability of recurrence and survival in early-stage LUAD.

Additionally, ICIs targeting both PD1 and PD-L1 have achieved great advances in the multidisciplinary treatment of lung cancer in recent years (53). Our signature was also revealed to be correlated with tumor immune activity. Hence, we performed an analysis to describe the immune checkpoint profile and made attempts to predict the immunotherapy response. Using the TIDE algorithm, we found that the high-risk group patients had high TMB, PD-L1 expression, and neoantigen burden, which have been proven to be useful immunotherapy biomarkers (20, 21, 31). These findings preliminarily indicated that high-risk patients may benefit from ICIs. Moreover, the TIDE score, a more accurate predictor of immunotherapy response than TMB, PD-L1 expression, or neoantigen burden (22), was found to be decreased in patients in the high-risk group. This finding suggested that the high-risk patients were characterized by tumor immune evasion potential and were more likely to benefit from ICIs, which is inconsistent with the above results. The above interesting findings demonstrated that high- and low-risk patients had diverse immune checkpoint profiles, and our LMRG-based signature could identify those who were suitable for treatment with checkpoint inhibitors.

Notably, we further conducted an analysis to predict chemotherapy response to understand the role of our signature in early-stage LUAD. The results showed that the estimated IC_50_ for each of four conventionally used drugs was significantly higher in the low-risk group than in the high-risk group, indicating that the LUAD patients with higher risk scores tended to be more sensitive to chemotherapy. This finding indicated that our signature could be applied for personalized treatment in LUAD patients. Oren (13) found that an upregulated fatty acid oxidation level was associated with a persistent proliferative capacity across multiple cancer types. This finding explains the higher chemoresistance of the low-risk group, which was characterized by significantly enriched fatty acid metabolism.

Although our LMRG-based signature showed promise for the prediction of cancer recurrence, as well as immunotherapy and chemotherapy response, limitations of the study exist. First, all the analyzed cohorts were from retrospective public databases; hence, prospective validation of our results in fresh specimens is needed. Second, the results of the TIIC landscape and immune checkpoint profile analyses were estimated from transcriptomic data; hence, the correlation between our signature and immunotherapy response still warrants further verification in future immunotherapy cohorts.

In summary, for the first time, we established and described a novel recurrence-associated LMRG-based signature for patients with early-stage LUAD. The LMRG-based signature developed in this study has the potential to be used as an effective predictor of immune checkpoint inhibitor and drug response to achieve individualized antitumor treatment by identifying those patients who may benefit from immunotherapy and chemotherapy.

## Data Availability Statement

Publicly available datasets were analyzed in this study. This data can be found here: TCGA database (https://portal.gdc.cancer.gov/), and GEO database (https://www.ncbi.nlm.nih.gov/geo/).

## Author Contributions

JH conceived, designed, and supervised the study. MZ and QZ collected, analyzed, and visualized the data. TF, YL, FW, SZ, XW, and HZ provided support for the statistical analysis and results interpretation. MZ and QZ wrote the draft of the manuscript. JH, QX, NS, and FT revised and edited the manuscript. All authors contributed to the article and approved the submitted version.

## Funding

This work was supported by the National Key Basic Research Development Plan (2018YFC1312105), CAMS Innovation Fund for Medical Sciences (2017-I2M-1-005) and Beijing Natural Science Foundation (J20010).

## Conflict of Interest

The authors declare that the research was conducted in the absence of any commercial or financial relationships that could be construed as a potential conflict of interest.

## Publisher’s Note

All claims expressed in this article are solely those of the authors and do not necessarily represent those of their affiliated organizations, or those of the publisher, the editors and the reviewers. Any product that may be evaluated in this article, or claim that may be made by its manufacturer, is not guaranteed or endorsed by the publisher.

## Supplementary Material

Supplementary Figure 1 The overall workflow of the signature construction and validation for the current study.

Supplementary Figure 2 Consensus clustering results of different validation groups by their optimal cluster number separately. And Kaplan–Meier curve survival analysis of patients stratified by cluster subtype in validation groups.

Supplementary Figure 3 Survival difference between the two risk groups in subsets stratified by age, sex, smoking history, and EGFR, KRAS, and ALK mutation status in the TCGA cohort.

Supplementary Figure 4 Prognostic meta-analysis results in the stage I **(A)** and stage II **(B)** disease subsets based on the TCGA and GEO datasets.

Supplementary Figure 5 Validation of the LMRG-based signature in different GEO cohorts. **(A–D)** Kaplan-Meier curves of OS in different GEO cohorts based on risk score. **(E)** Results of the prognostic meta-analysis based on the TCGA and GEO datasets.

Supplementary Figure 6 Waterfall plots showing the top mutated genes in the high-risk group **(A)** and low-risk group **(B)**.

Supplementary Figure 7 Immune microenvironment profiles related to the LMRG-based signature. The estimated LAG3 **(A)**, TIM3 **(B)**, TAM M2 score **(C)** MDSC score **(D)**, CAF score **(E)**, and CD8 score **(F)** in the two risk groups are shown. The correlations between risk score and the estimated T cell exclusion **(G)** and the T cell dysfunction (H) levels are shown.
