# Corrigendum: Clinical Significance and Immunometabolism Landscapes of a Novel Recurrence-Associated Lipid Metabolism Signature In Early-Stage Lung Adenocarcinoma: A Comprehensive Analysis

**DOI:** 10.3389/fimmu.2022.909105

**Published:** 2022-05-04

**Authors:** Mingchuang Zhu, Qingpeng Zeng, Tao Fan, Yuanyuan Lei, Feng Wang, Sufei Zheng, Xinfeng Wang, Hui Zeng, Fengwei Tan, Nan Sun, Qi Xue, Jie He

**Affiliations:** ^1^ Department of Oncology, Renmin Hospital of Wuhan University, Wuhan, China; ^2^ National Cancer Center/National Clinical Research Center for Cancer/Cancer Hospital, Chinese Academy of Medical Sciences and Peking Union Medical College, Beijing, China

**Keywords:** lipid metabolism, early-stage lung adenocarcinoma (LUAD), recurrence, immune checkpoints(ICP), signature

In the published article, there was an error regarding the affiliations for author Jie He. As well as having affiliation 2, he should also have affiliation 1.

The authors apologize for this error and state that this does not change the scientific conclusions of the article in any way. The original article has been updated.

A correction has been made to the Abstract, subsection Background, paragraph one:

In the original article, there was an error. The sentence “The early-stage lung adenocarcinoma (LUAD) incidence has increased with heightened public awareness and lung cancer screening implementation” has been corrected to

“The early-stage lung adenocarcinoma (LUAD) rate has increased with heightened public awareness and lung cancer screening implementation.”

A correction has been made to the Introduction, paragraph one:

In the original article, there was an error. The sentence “The incidence of early-stage LUAD has increased rapidly with heightened public awareness and implementation of lung cancer screening” has been corrected to

“The rate of early-stage LUAD has increased rapidly with heightened public awareness and implementation of lung cancer screening”.

The authors apologize for these errors and state that this does not change the scientific conclusions of the article in any way. The original article has been updated.

## Publisher’s Note

All claims expressed in this article are solely those of the authors and do not necessarily represent those of their affiliated organizations, or those of the publisher, the editors and the reviewers. Any product that may be evaluated in this article, or claim that may be made by its manufacturer, is not guaranteed or endorsed by the publisher.

