# Supplementary figures and images for: Clinical Significance and Immunometabolism Landscapes of a Novel Recurrence-Associated Lipid Metabolism Signature In Early-Stage Lung Adenocarcinoma: A Comprehensive Analysis
Source: Front Immunol. 2022 Feb 10;13:783495. doi: 10.3389/fimmu.2022.783495 (PMC8867215; doi:10.3389/fimmu.2022.783495)

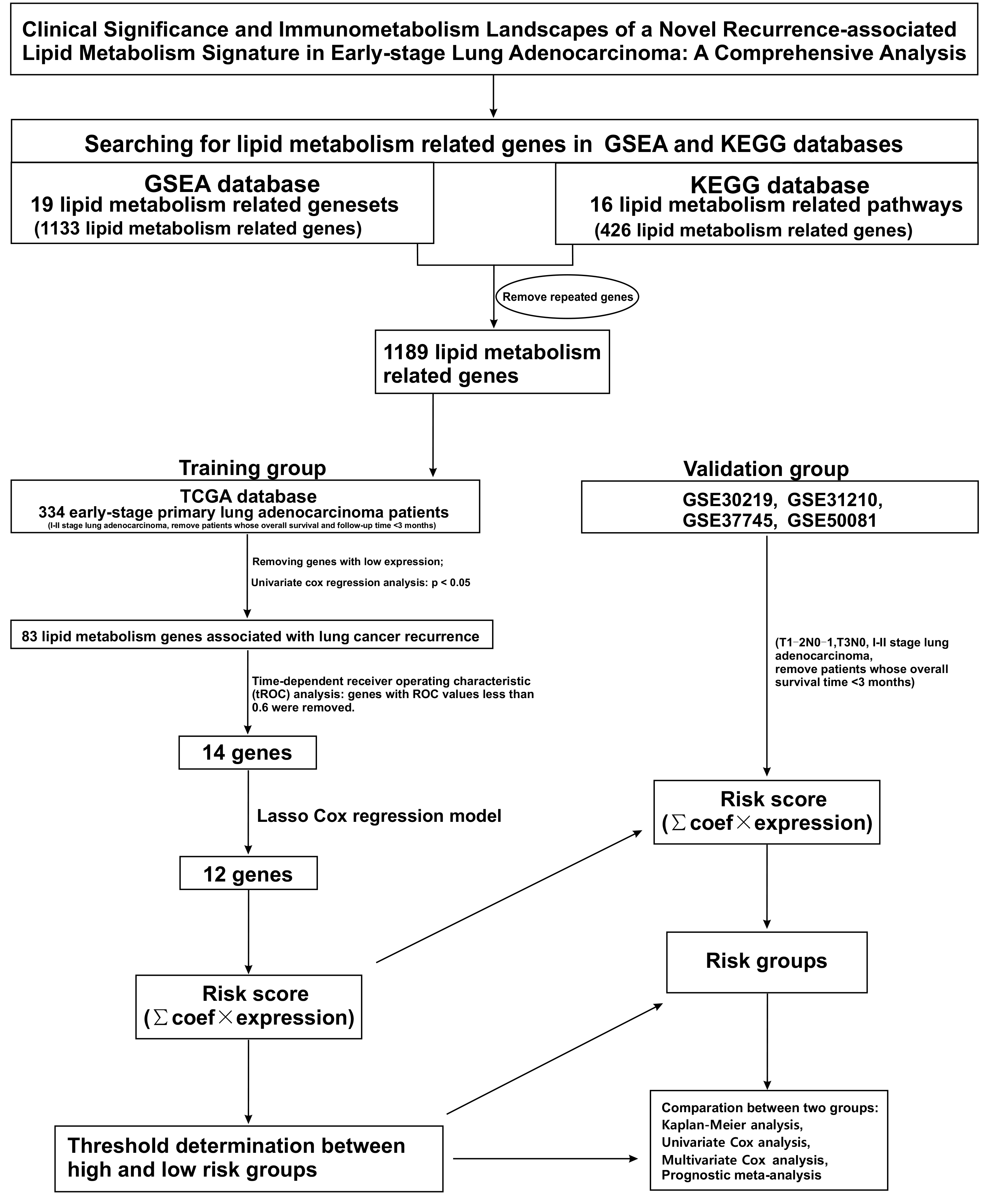

Supplement: Supplementary Figure 1 — The overall workflow of the signature construction and validation for the current study. [file Image_1.tif]

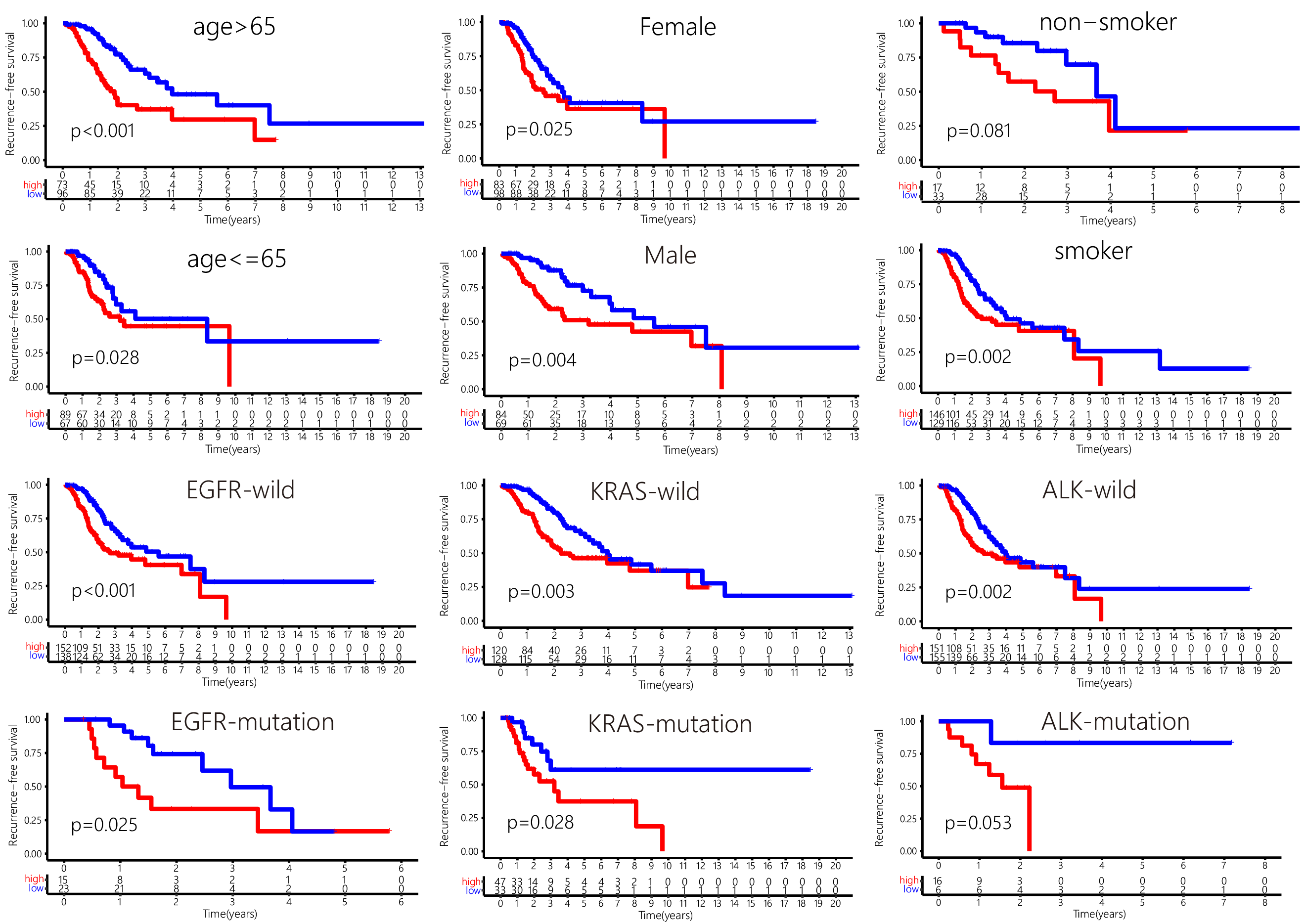

Supplement: Supplementary Figure 3 — Survival difference between the two risk groups in subsets stratified by age, sex, smoking history, and EGFR, KRAS, and ALK mutation status in the TCGA cohort. [file Image_3.tif]

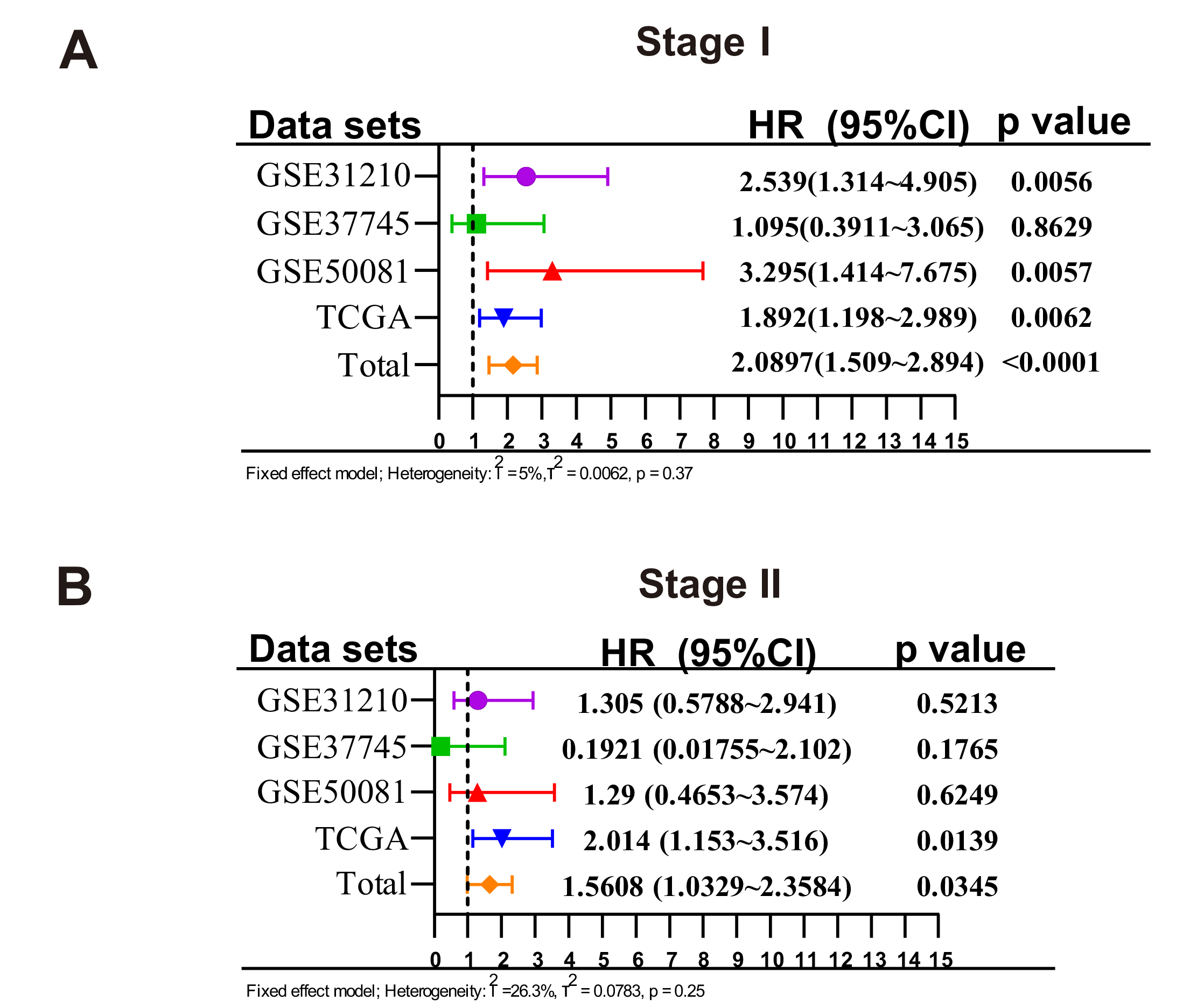

Supplement: Supplementary Figure 4 — Prognostic meta-analysis results in the stage I (A) and stage II (B) disease subsets based on the TCGA and GEO datasets. [file Image_4.tif]

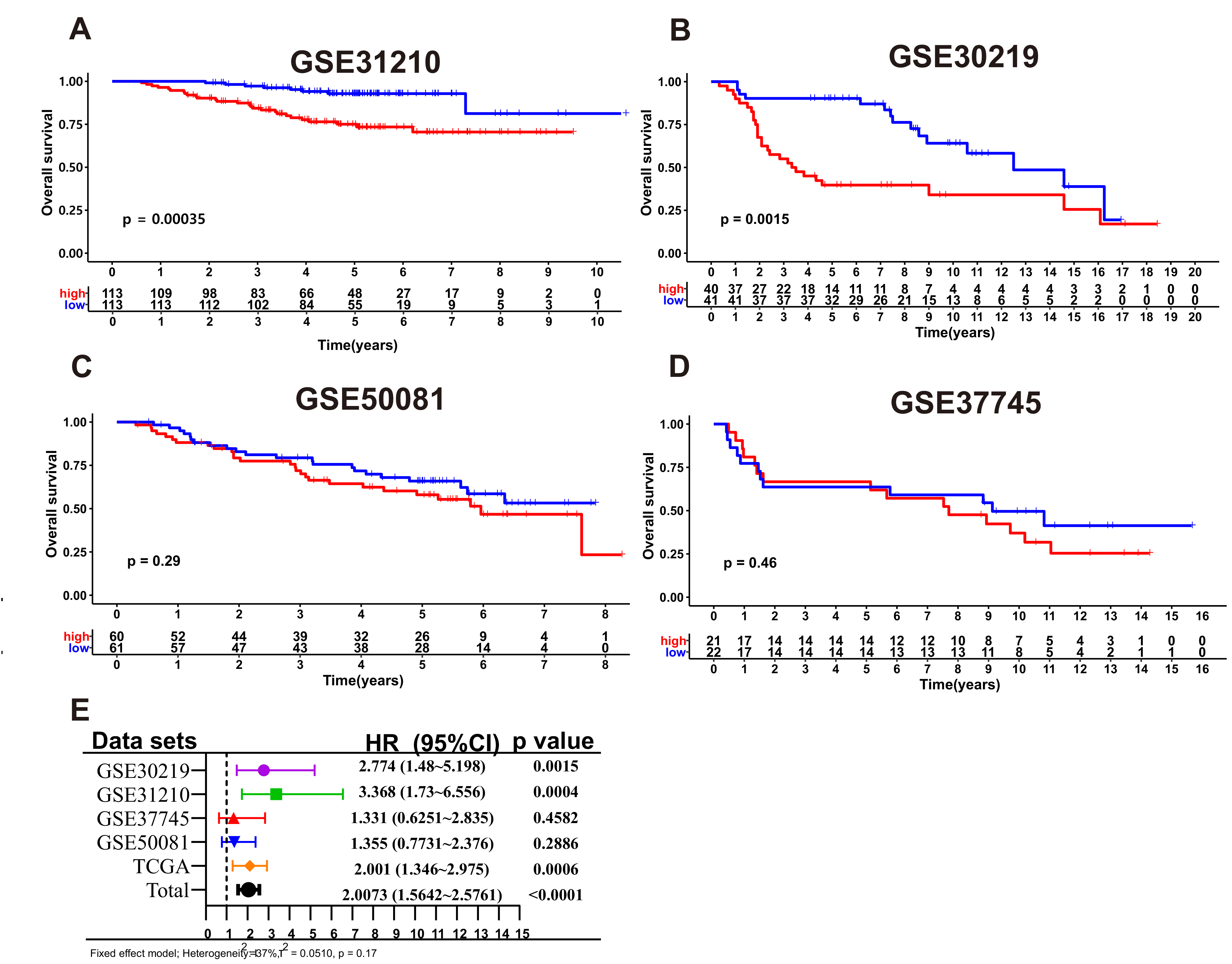

Supplement: Supplementary Figure 5 — Validation of the LMRG-based signature in different GEO cohorts. (A–D) Kaplan-Meier curves of OS in different GEO cohorts based on risk score. (E) Results of the prognostic meta-analysis based on the TCGA and GEO datasets. [file Image_5.tif]

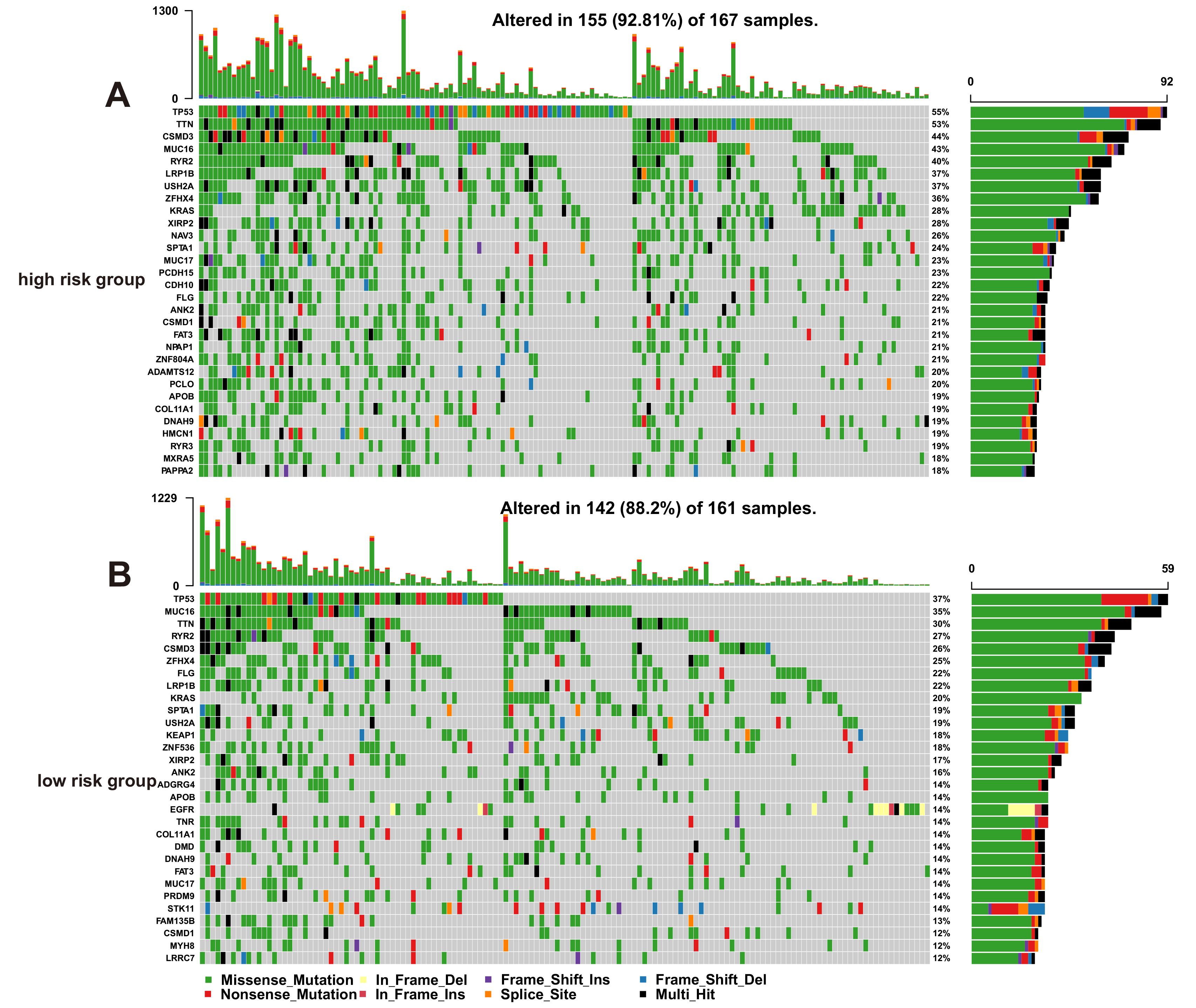

Supplement: Supplementary Figure 6 — Waterfall plots showing the top mutated genes in the high-risk group (A) and low-risk group (B). [file Image_6.tif]

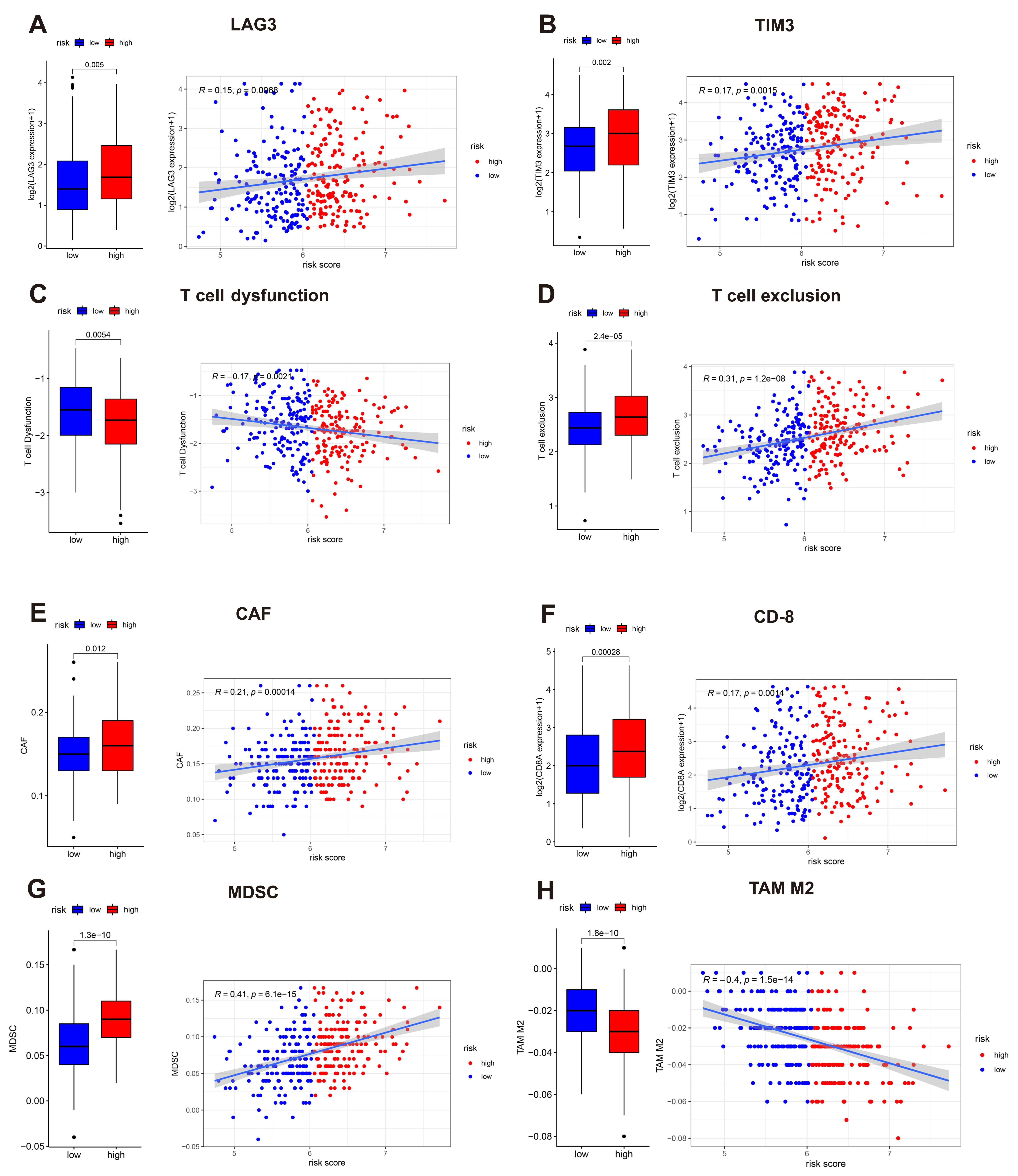

Supplement: Supplementary Figure 7 — Immune microenvironment profiles related to the LMRG-based signature. The estimated LAG3 (A), TIM3 (B), TAM M2 score (C) MDSC score (D), CAF score (E), and CD8 score (F) in the two risk groups are shown. The correlations between risk score and the estimated T cell exclusion (G) and the T cell dysfunction (H) levels are shown. [file Image_7.tif]
