# Supplementary material for: Clinical Significance and Immunometabolism Landscapes of a Novel Recurrence-Associated Lipid Metabolism Signature In Early-Stage Lung Adenocarcinoma: A Comprehensive Analysis
Source: Front Immunol. 2022 Feb 10;13:783495. doi: 10.3389/fimmu.2022.783495 (PMC8867215; doi:10.3389/fimmu.2022.783495)
Supplement: Supplementary file 8 [file Table_1.docx]

**Supplementary Tables**

**Supplementary Figure Legends**

**Figure S1.** The overall workflow of the signature construction and validation for the current study.

**Figure S2.** Consensus clustering results of different validation groups by their optimal cluster number separately. And Kaplan–Meier curve survival analysis of patients stratified by cluster subtype in validation groups.

**Figure S3.** Survival difference between the two risk groups in subsets stratified by age, sex, smoking history, and EGFR, KRAS, and ALK mutation status in the TCGA cohort.

**Figure S4.** Prognostic meta-analysis results in the stage I (A) and stage II (B) disease subsets based on the TCGA and GEO datasets.

**Figure S5.** Validation of the LMRG-based signature in different GEO cohorts. A-D, Kaplan-Meier curves of OS in different GEO cohorts based on risk score. E, Results of the prognostic meta-analysis based on the TCGA and GEO datasets.

**Figure S6.** Waterfall plots showing the top mutated genes in the high-risk group (A) and low-risk group (B).

**Figure S7.** Immune microenvironment profiles related to the LMRG-based signature. The estimated LAG3 (A), TIM3 (B), TAM M2 score (C) MDSC score (D), CAF score (E), and CD8 score (F) in the two risk groups are shown. The correlations between risk score and the estimated T cell exclusion (G) and the T cell dysfunction (H) levels are shown.

**Supplementary Table S1.** The detailed lipid metabolism-related gene sets from the GSEA and KEGG database.

| **Database** | **gene-set** |
| --- | --- |
| **GSEA** | HALLMARK_FATTY_ACID_METABOLISM |
|  | KEGG_FATTY_ACID_METABOLISM |
|  | KEGG_GLYCEROLIPID_METABOLISM |
|  | KEGG_GLYCEROPHOSPHOLIPID_METABOLISM |
|  | KEGG_SPHINGOLIPID_METABOLISM |
|  | REACTOME_FATTY_ACID_METABOLISM |
|  | REACTOME_GLYCOSPHINGOLIPID_METABOLISM |
|  | REACTOME_METABOLISM_OF_LIPIDS |
|  | REACTOME_REGULATION_OF_LIPID_METABOLISM_LISM_BY_PPARALPHA |
|  | WP_CHOLESTEROL_METABOLISM_INCLUDES_BOTH_BOTH_BLOCH_AND_KANDUTSCHRUSSELL_PATHWAYS |
|  | REACTOME_SPHINGOLIPID_METABOLISM |
|  | WP_LIPID_METABOLISM_PATHWAY |
|  | PHOSPHOLIPID_METABOLIC_PROCESS |
|  | HALLMARK_FATTY_ACID_METABOLISM |
|  | REACTOME_KETONE_BODY_METABOLISM |
|  | GO_REGULATION_OF_CHOLESTEROL_METABOLIC_PLIC_PROCESS |
|  | REACTOME_PHOSPHOLIPID_METABOLISM |
|  | REACTOME_REGULATION_OF_LIPID_METABOLISM_LISM_BY_PPARALPHA |
|  | REACTOME_TRANSCRIPTIONAL_REGULATION_OF_W_OF_WHITE_ADIPOCYTE_DIFFERENTIATION |
| **KEGG** | hsa00061 Fatty acid biosynthesis |
|  | hsa00062 Fatty acid elongation |
|  | hsa00071 Fatty acid degradation |
|  | hsa00072 Synthesis and degradation of ketone bodies |
|  | hsa00100 Steroid biosynthesis |
|  | hsa00120 Primary bile acid biosynthesis |
|  | hsa00140 Steroid hormone biosynthesis |
|  | hsa00561 Glycerolipid metabolism |
|  | hsa00564 Glycerophospholipid metabolism |
|  | hsa00565 Ether lipid metabolism |
|  | hsa00590 Arachidonic acid metabolism |
|  | hsa00591 Linoleic acid metabolism |
|  | hsa00592 alpha-Linolenic acid metabolism |
|  | hsa00600 Sphingolipid metabolism |
|  | hsa01040 Biosynthesis of unsaturated fatty acids |
|  | hsa04979 Cholesterol metabolism |

**Supplementary Table S2.** Univariate Cox regression results of the 83-lipid metabolism-related gene with cancer recurrence.

| **id** | **HR** | **HR.95L** | **HR.95H** | ***P* value** |
| --- | --- | --- | --- | --- |
| CES1 | 0.891488 | 0.810868 | 0.980123 | 0.017544 |
| ELOVL6 | 1.27791 | 1.054583 | 1.54853 | 0.012341 |
| ERLIN2 | 1.392962 | 1.037721 | 1.869811 | 0.027351 |
| GPAM | 0.690401 | 0.501046 | 0.951318 | 0.023507 |
| ACADS | 0.726038 | 0.562083 | 0.937818 | 0.014223 |
| ACOX1 | 1.513356 | 1.026974 | 2.230094 | 0.036214 |
| ADIPOR2 | 1.417927 | 1.004909 | 2.000695 | 0.046829 |
| AQP7 | 0.780101 | 0.610353 | 0.997058 | 0.047312 |
| DLST | 1.675064 | 1.109777 | 2.528293 | 0.014056 |
| EHHADH | 1.442869 | 1.032482 | 2.016373 | 0.03178 |
| EPHX1 | 0.782139 | 0.679332 | 0.900504 | 0.000632 |
| GCDH | 0.591813 | 0.376772 | 0.92959 | 0.022794 |
| GPD1 | 0.731042 | 0.570881 | 0.936137 | 0.013028 |
| GPD2 | 1.550801 | 1.101699 | 2.182979 | 0.011898 |
| INMT | 0.8748 | 0.767525 | 0.997068 | 0.045075 |
| LDHA | 1.618685 | 1.242583 | 2.108624 | 0.000357 |
| MLYCD | 0.423402 | 0.217558 | 0.824004 | 0.011414 |
| NBN | 1.370034 | 1.020017 | 1.84016 | 0.036471 |
| NSDHL | 1.443361 | 1.002685 | 2.077712 | 0.048337 |
| RETSAT | 1.463491 | 1.063473 | 2.013973 | 0.019401 |
| SMS | 1.389445 | 1.043425 | 1.850212 | 0.024394 |
| TP53INP2 | 1.258474 | 1.007972 | 1.571231 | 0.042349 |
| UROS | 0.586769 | 0.36528 | 0.942559 | 0.027483 |
| INPP4B | 1.344485 | 1.067779 | 1.692896 | 0.01181 |
| INPP5E | 0.710114 | 0.50428 | 0.999964 | 0.049976 |
| MBOAT2 | 1.263422 | 1.016034 | 1.571047 | 0.035463 |
| MFSD2A | 0.842666 | 0.715276 | 0.992745 | 0.040654 |
| OSBPL10 | 1.468064 | 1.046955 | 2.058553 | 0.026014 |
| PITPNB | 1.617673 | 1.004741 | 2.604518 | 0.047768 |
| PLA2G4F | 0.807685 | 0.668409 | 0.975982 | 0.026987 |
| PLD1 | 1.428134 | 1.001232 | 2.037058 | 0.049211 |
| PNPLA8 | 1.559201 | 1.055359 | 2.303583 | 0.025711 |
| RAB5A | 1.946495 | 1.211191 | 3.128194 | 0.005932 |
| SBF2 | 1.443939 | 1.05019 | 1.985317 | 0.023734 |
| SLC44A1 | 1.417962 | 1.035909 | 1.940918 | 0.029241 |
| ABHD5 | 1.463565 | 1.011837 | 2.116963 | 0.04313 |
| ACSL3 | 1.42304 | 1.092854 | 1.852986 | 0.008815 |
| FITM1 | 0.331968 | 0.127991 | 0.861017 | 0.023348 |
| FLT1 | 1.296477 | 1.028289 | 1.63461 | 0.0281 |
| GPLD1 | 0.450315 | 0.223509 | 0.907271 | 0.025599 |
| HTR2C | 1.430073 | 1.017945 | 2.009056 | 0.039159 |
| IMPA1 | 1.40926 | 1.014835 | 1.956982 | 0.040573 |
| INPP5B | 0.612947 | 0.43037 | 0.87298 | 0.006671 |
| IRS1 | 1.275511 | 1.051331 | 1.547494 | 0.013605 |
| SGPP1 | 1.34729 | 1.047438 | 1.732981 | 0.020298 |
| SLC27A1 | 0.635248 | 0.466365 | 0.865289 | 0.004007 |
| WASHC1 | 0.712145 | 0.511897 | 0.990728 | 0.043877 |
| WDR91 | 0.725884 | 0.549485 | 0.95891 | 0.024112 |
| ACOXL | 0.676695 | 0.500556 | 0.914815 | 0.011125 |
| CYP2C8 | 0.426285 | 0.214031 | 0.849031 | 0.015286 |
| CYP4B1 | 0.904743 | 0.833089 | 0.98256 | 0.017413 |
| DPEP2 | 0.718163 | 0.536523 | 0.961296 | 0.026062 |
| ELOVL7 | 1.287119 | 1.048006 | 1.580788 | 0.016078 |
| HACD2 | 1.414707 | 1.023022 | 1.956356 | 0.035942 |
| HPGDS | 0.779705 | 0.636519 | 0.955101 | 0.01623 |
| HSD17B3 | 0.37605 | 0.17205 | 0.821937 | 0.014227 |
| PRKAG3 | 0.000371 | 2.74E-07 | 0.50299 | 0.031814 |
| ACER1 | 0.00818 | 0.000117 | 0.572601 | 0.026611 |
| ASAH2 | 4.873932 | 1.29223 | 18.38312 | 0.019363 |
| SGPP2 | 1.228929 | 1.048385 | 1.440564 | 0.010996 |
| SPTSSA | 1.195143 | 1.001672 | 1.425982 | 0.047872 |
| SPTSSB | 1.171559 | 1.002229 | 1.369498 | 0.046821 |
| SUMF1 | 1.495195 | 1.034528 | 2.160993 | 0.032306 |
| CYP27A1 | 0.818677 | 0.695293 | 0.963956 | 0.016377 |
| ALDH2 | 0.748894 | 0.618398 | 0.906928 | 0.003076 |
| AGPS | 1.712005 | 1.170909 | 2.50315 | 0.005537 |
| ANGPTL4 | 1.156471 | 1.038681 | 1.287618 | 0.007992 |
| FHL2 | 1.221026 | 1.037479 | 1.437045 | 0.016276 |
| GSTM4 | 0.676549 | 0.470202 | 0.973452 | 0.035299 |
| HSD17B13 | 0.703165 | 0.540221 | 0.915258 | 0.008836 |
| MED6 | 1.849288 | 1.169757 | 2.923567 | 0.008515 |
| PLIN3 | 1.414124 | 1.070197 | 1.868577 | 0.014804 |
| PPP1CB | 1.574397 | 1.111654 | 2.229763 | 0.010586 |
| SEC23A | 1.39412 | 1.10974 | 1.751375 | 0.00431 |
| TBL1XR1 | 1.540693 | 1.114369 | 2.130116 | 0.008919 |
| ADH1B | 0.893624 | 0.800363 | 0.997752 | 0.045503 |
| CCND3 | 0.80709 | 0.653258 | 0.997148 | 0.046985 |
| KLF5 | 1.271923 | 1.04275 | 1.551464 | 0.017647 |
| PCK1 | 1.426806 | 1.12657 | 1.807058 | 0.003192 |
| DGKK | 2.304724 | 1.187046 | 4.474768 | 0.013645 |
| VDAC1 | 1.659784 | 1.186946 | 2.320985 | 0.003059 |
| SULT2B1 | 1.266162 | 1.074782 | 1.491619 | 0.004765 |
| GPX3 | 0.763797 | 0.648753 | 0.899242 | 0.001216 |
